# Interaction of Arsenic with Zinc and Organics in a Rice (*Oryza sativa* L.)—Cultivated Field in India

**DOI:** 10.1100/tsw.2005.60

**Published:** 2005-08-18

**Authors:** Dilip Kumar Das, T. K. Garai, S. Sarkar, Pintu Sur

**Affiliations:** ^1^ Department of Agricultural Chemistry and Soil Science, Bidhan Chandra Krishi Viswavidyalaya, Mohanpur 741 252, Nadia, West Bengal, India; ^2^ Department of Agricultural Meteorology and Physics, Bidhan Chandra Krishi Viswavidyalaya, Mohanpur 741 252, Nadia, West Bengal, India

**Keywords:** arsenic, interaction, organics, rice soil, zinc

## Abstract

A laboratory experiment on an Inceptisol with pH 7.6, organic carbon 6.8 g kg^-1^, and 0.5 *M* NaHCO_3_ extractable arsenic 0.4 mg kg^-1^ was conducted to study the interaction effect of graded levels of arsenic (0, 5, and 10 mg kg^-1^) with zinc (0, 10, and 20 mg kg^-1^) and organics (0, 1, and 2% on soil weight basis) separately on the mobilization of arsenic in soils.The results show that the amount of 0.5 *M* NaHCO_3_ extractable arsenic at pH 8.5 increased with the progress of submergence up to 35 days. However, the increase in arsenic concentration was correlated with decreasing application of graded levels of Zn as zinc sulfate. The intensity of reduction varied with varying levels of Zn, being higher (0.73—2.72 mg kg^-1^) in the treatment where Zn was at 10 mg kg^-1^ and lower (0.70—1.08 mg kg^-1^) with Zn at 20 mg kg^-1^ application.The amount of arsenic content in the soil significantly decreased with the application of varying levels of organics. However, such depressive effect was found more pronounced with well-decomposed farm yard manure than that of vermicompost. The results of field experiments showed that the grain yield between continuous flooding (4.84 t ha^-1^) and intermittent flooding up to 40 days after transplanting then continuous flooding (4.83 t ha^-1^) with the application of ZnSO_4_ at 25 kg ha^-1^ did not vary. The lowest grain yield (3.65 t ha^-1^) was recorded in the treatment where intermittent flooding was maintained throughout the growth period without the application of Zn. The amount of arsenic content was, however, recorded much lower in the treatment where intermittent flooding throughout the growth period was maintained with ZnSO_4_.

## INTRODUCTION

In Asian countries, the highest number of people consuming and using arsenic-contaminated drinking water and groundwater for irrigation purposes suffer from arsenicosis, which has been reported by Chakravarty and Das[1]. Out of nineteen (19) districts in West Bengal, recently nine (9) districts are arsenic affected where cropping intensities are very high. For the agricultural crop production, particularly boro (summer) rice, during the lean period (i.e., March to May, the period for scarcity of water), a large amount of groundwater loaded with arsenic is used for irrigating agricultural crops.

Mandal[2] reported that 8–9 million people in West Bengal are affected by arsenic toxicities due to consuming such arsenic-contaminated groundwater for domestic use. The average arsenic contamination in the drinking water is about 0.20 mg $l^{-1}$ with a maximum concentration of 3.7 mg $l^{-1}$. Based on World Health Organization (WHO) specification (0.05 mg $l^{-1}$), about 45% of the tested drinking water is toxic. Chakravarty and Das[1] also reported that due to the use of such water for irrigation purposes, arsenic concentration occurs in the soil and subsequently enters into different parts of crops (including the economic products) and ultimately enters into humans and animals, causing various anomalies and carcinogenesis.

Rice, a main cereal crop in South East Asian countries, is especially susceptible to arsenic toxicity compared to upland crop plants because of the increase in both the availability and toxicity of arsenic under the reducing condition of submerged soils. Arsenic pollution in soil may have toxic effects in vegetation and can adversely affect the animals that feed on that vegetation. Phytotoxicity studies have shown that 7 ppm soluble arsenic causes injury to rice[3] and 9 ppm to peas, beans, and barley[4]. It has also been reported that the toxicity of arsenic may be reduced by applying sulfates of zinc, iron, and aluminum to the soil[5]. Very limited research has so far been carried out about the interaction of arsenic with Fe, Zn, and organic matter in soils in relation to rice.

Keeping this in mind, the present study was undertaken to investigate the interaction of arsenic with Zn and organics in the soil cultivated with rice (*Oryza sativa* L.). The objective of our research was to optimize water use efficiency with a view to minimize arsenic buildup in soil vis-à-vis absorption by rice without the yield reduction.

## EXPERIMENTAL METHODS

A laboratory experiment was conducted in an Aeric Endoaquepts (with pH 7.6, 0.5 *M* $NaHCO_3$ extractable arsenic of 0.22 mg $kg^{-1}$) to study the influence of Zn and organic matter on arsenic mobility in the soil. Arsenic doses were 0 ($As_0$), 5 ($As_1$), and 10 ($As_2$) mg $kg^{-1}$; Zn doses were 0 ($Zn_0$), 10 ($Zn_1$), and 20 ($Zn_2$) mg $kg^{-1}$; and organic matter (O) well-decomposed farm yard manure (FYM) and vermicompost (V) each at 0 ($O_0$ and $V_0$), 1 ($O_1$ and $V_1$), and 2% ($O_2$ and $V_2$) on weight basis were applied to all possible treatment combinations replicated thrice in a completely randomized block design. The effect of Zn and organic matter with arsenic were studied separately.

A field experiment was conducted on an arsenic-contaminated Aeric Endoaquept soil of Gotera village in the district of Nadia, India with summer rice (cv. IET 4786) as a test crop having soil physicochemical properties of pH 7.6, 0.5 *M* $NaHCO_3$ extractable arsenic 1.48 mg $kg^{-1}$. The experimental plot (30 × 17.5 $m^2$) was divided into 12 main plots (6 × 4.5 $m^2$) with each main plot being further subdivided into 2 subplots (3 × 4.5 $m^2$) in a split-plot design with four replicates. Recommended levels of 100, 50, and 50 kg $ha^{-1}$ N, $P_2O_5$, and $K_2O$ were applied to each subplot, where half of the N and full $P_2O_5$ and $K_2O$ were applied as basal and one-fourth of the N was top dressed at active tillering and panicle initiation stage. Two to three 30-day-old seedlings were transplanted at a spacing of 15 × 15 cm. The source of irrigation water was shallow-tube well containing 0.26 mg $l^{-1}$ arsenic. The main plot treatments received three levels of irrigation water namely (1) continuous ponding ($I_0$), (2) intermittent ponding ($I_1$), and (3) intermittent ponding up to 40 days of crop growth and then continuous ponding for the entire crop growth period ($I_2$). The subplot treatment was two levels of Zn ($Zn_0$ and $Zn_1$, at 25 kg $ha^{-1}$ $ZnSO_4$). Then rice was allowed to grow until the harvest. Soil and plant samples were collected periodically and also at harvest for the estimation of the arsenic content in both soils and plants. Arsenic was determined with the help of an atomic absorption spectrophotometer after extracting soils with 0.5 *M* $NaHCO_3$ at pH 8.5[6] and plant samples were digested with ternary acid mixture of $HClO_4$:$HNO_3$:$H_2SO_4$ (10:4:1)[7].

## RESULTS AND DISCUSSION

The results (Table 1) show that a slight increase in the amount of arsenic content was found in native soil due to submergence in an absolute control treatment ($As_0Zn_0$). The amount of arsenic content in the soil significantly decreased with the application of Zn; the highest recorded decrease (38.4%) with 10 mg kg$^{-1}$ Zn compared to 20 mg kg$^{-1}$ (31.5%) Zn application after 35 days of submergence. The results also show that the amount of arsenic content consistently increased with the progress of submergence due to application of arsenic. The rate of increase, however, was recorded lower and higher at the initial and later period of submergence, which might be due to varying intensity of reduction of arsenate to arsenite with the progress of submergence. Mukhopadhyay et al.[8] reported that after 14 days of submergence, soil had Eh values of –115 mV and, under these conditions, arsenate was reduced to arsenite. Arsenite, a more toxic form of arsenic, would be an important factor in the low Eh and pH levels of a flooded soil system.

**TABLE 1**
**Effect of Zn and Arsenic Application on the Mobilization of Arsenic (mg kg$^{-1}$) in a Submerged Soil**

| | Days after Submergence | | | | | | | | | | | | | | | | | | | |
|---|---|---|---|---|---|---|---|---|---|---|---|---|---|---|---|---|---|---|---|---|
| Treatments | 7 | | | | 14 | | | | 21 | | | | 28 | | | | 35 | | | |
| | $As_0$ | $As_1$ | $As_2$ | Mean | $As_0$ | $As_1$ | $As_2$ | Mean | $As_0$ | $As_1$ | $As_2$ | Mean | $As_0$ | $As_1$ | $As_2$ | Mean | $As_0$ | $As_1$ | $As_2$ | Mean |
| $Zn_0$ | 0.23 | 2.35 | 2.48 | 1.68 | 0.31 | 2.98 | 2.79 | 2.02 | 0.34 | 3.17 | 3.87 | 2.46 | 0.38 | 3.42 | 4.16 | 2.65 | 0.36 | 3.49 | 4.36 | 2.73 |
| $Zn_1$ | 0.16 | 1.27 | 1.43 | 0.95 | 0.20 | 1.33 | 1.48 | 1.01 | 0.25 | 1.60 | 1.89 | 1.24 | 0.28 | 1.92 | 2.21 | 1.47 | 0.30 | 2.22 | 2.48 | 1.68 |
| $Zn_2$ | 0.19 | 1.22 | 1.53 | 0.98 | 0.24 | 1.51 | 1.68 | 1.14 | 0.29 | 1.78 | 2.07 | 1.38 | 0.32 | 2.11 | 2.41 | 1.61 | 0.35 | 2.39 | 2.88 | 1.87 |
| Mean | 0.19 | 1.61 | 1.81 | | 0.25 | 1.94 | 1.98 | | 0.29 | 2.18 | 2.61 | | 0.32 | 2.48 | 2.92 | | 0.33 | 2.71 | 3.24 | |
| CD ($P$ = 0.05) | 0.14 | 0.21 | 0.24 | | 0.16 | 0.30 | 0.25 | | 0.27 | 0.32 | 0.28 | | 0.18 | 0.33 | 0.42 | | 0.19 | 0.41 | 0.39 | |

With regard to interaction between Zn and arsenic, it was found that the amount of available arsenic content decreased; maximum amount with the $Zn_{10}As_5$ treatment combination, although the concentration of arsenic was minimum in all treatments with Zn and without the addition of arsenic. The results, therefore, confirmed that the Zn has pronounced effect in reducing the available arsenic concentration in a submerged soil[9].

Tables 2 and 3 show that the amount of arsenic content decreases with the increase of organic matter application, irrespective of sources. The magnitude of such decreases, however, varied with sources and levels of applied organic matter; greater decrease (18.30%) with higher levels of vermicompost (2% by weight of soil) application compared to corresponding levels of well-decomposed organic matter (14.01%) after 50 days of submergence. The same trend was followed for the changes of arsenic concentration in the soil due to application of arsenic where the concentration of the same increased up to 25 days of submergence and thereafter its concentration decreased at 50 days of submergence.

Considering the interaction effect between vermicompost and arsenic, it was recorded that the amount of arsenic content decreases irrespective of treatments. The highest decrease was recorded in $As_0V_1$ and $As_0V_2$ treatment combinations where the arsenic was not applied. The decrease was maximum with $As_1O_2$ treatment combination where arsenic at 5 mg kg$^{-1}$ was applied together with 2% vermicompost by weight of soil, which might be due to formation of insoluble arseno-organic complexes and its adsorption on to organic colloids[10].

**TABLE 2**
**Effect of Well-Decomposed Organic Matter on the Mobilization of Arsenic (mg $kg^{-1}$) in the Soil**

| Treatments | Arsenic Concentration (mg $kg^{-1}$) at Different Days of Incubation | | | | | | | | | | | |
|---|---|---|---|---|---|---|---|---|---|---|---|---|
| | 10 Days | | | | 25 Days | | | | 50 Days | | | |
| | $As_0$ | $As_1$ | $As_2$ | Mean | $As_0$ | $As_1$ | $As_2$ | Mean | $As_0$ | $As_1$ | $As_2$ | Mean |
| $O_0$ | 2.05 | 2.58 | 3.73 | 2.78 | 2.89 | 3.32 | 4.14 | 3.45 | 2.63 | 3.13 | 3.89 | 3.10 |
| $O_1$ | 2.09 | 2.50 | 3.74 | 2.77 | 2.73 | 3.15 | 3.87 | 3.25 | 2.43 | 3.87 | 3.78 | 3.03 |
| $O_2$ | 2.15 | 2.43 | 3.66 | 2.74 | 2.67 | 3.23 | 3.78 | 3.22 | 2.12 | 2.63 | 3.53 | 2.76 |
| Mean | 2.09 | 2.50 | 3.71 | | 2.76 | 3.23 | 3.90 | | 2.39 | 2.88 | 3.73 | |
| CD ($p$ = 0.05) | 0.10 | 0.18 | 0.15 | | 0.12 | 0.14 | 0.15 | | 0.17 | 0.21 | 0.19 | |

**TABLE 3**
**Effect of Vermicompost on the Mobilization of Arsenic (mg $kg^{-1}$) in the Soil Studied**

| Treatments | Arsenic Concentration (mg $kg^{-1}$) at Different Days of Incubation | | | | | | | | | | | |
|---|---|---|---|---|---|---|---|---|---|---|---|---|
| | 10 Days | | | | 25 Days | | | | 50 Days | | | |
| | $As_0$ | $As_1$ | $As_2$ | Mean | $As_0$ | $As_1$ | $As_2$ | Mean | $As_0$ | $As_1$ | $As_2$ | Mean |
| $V_0$ | 2.13 | 2.48 | 3.89 | 2.83 | 2.76 | 3.51 | 4.18 | 3.48 | 2.59 | 3.23 | 3.71 | 3.17 |
| $V_1$ | 2.12 | 2.67 | 3.77 | 2.85 | 2.54 | 3.42 | 3.86 | 3.27 | 2.24 | 2.88 | 3.43 | 2.85 |
| $V_2$ | 2.16 | 2.42 | 3.64 | 2.74 | 2.69 | 3.59 | 3.71 | 3.33 | 2.01 | 2.65 | 3.13 | 2.59 |
| Mean | 2.13 | 2.52 | 3.76 | | 2.66 | 3.50 | 3.92 | | 2.28 | 2.91 | 3.42 | |
| CD ($p$ = 0.05) | 0.11 | 0.20 | 0.18 | | 0.24 | 0.17 | 0.21 | | 0.25 | 0.29 | 0.17 | |

Table 4 shows that the amount of arsenic buildup in the soil up to 75 days of submergence (3.98 mg $kg^{-1}$) significantly increased in the treatment where Zn was not applied. With regard to irrigation water management practices, it was recorded that the amount of arsenic buildup in the soil was found to be the highest (3.64 mg $kg^{-1}$) in $I_0$ treatment, followed by $I_1$ (3.58 mg $kg^{-1}$) and $I_2$ (2.75 mg $kg^{-1}$) irrespective of Zn applications. Such buildup of arsenic in the soil, however, has been found to be further counteracted by the application of $ZnSO_4$ at 25 kg $ha^{-1}$, as seen from the interaction effects between Zn and methods of applying irrigation water.

Considering the interaction effect between Zn and methods of water irrigation, the lowest amount of arsenic in the soil (1.98 mg $kg^{-1}$) was recorded on $Zn_1I_1$ treatment ($ZnSO_4$ at 25 kg $ha^{-1}$) with intermittent ponding up to 75 cm of crop growth. The arsenic toxicity may be reduced by applying sulfates of Zn, Fe, and Al to the soil[5,9]. Such decrease in its concentration might be due to the suppressing effect of Zn that results in precipitation/fixation of arsenic as Zn-arsenate, which makes it unavailable to plants[11].

$$\text{Arsenate} + ZnSO_4 \rightarrow \text{Zn-arsenate}$$

(Insoluble and unavailable to the plant)

The results further show that the accumulation of arsenic in different parts of rice plants varied with treatments in the following order:

Root > Stem > Leaf > Grain.

**TABLE 4**
**Effect of Arsenic-Contaminated Irrigation Water and Zn Fertilizers on the Mobility of Arsenic in Soil from a Field Cultivated with Rice (*O. sativa* L.) in India**

| Treatments | Arsenic Concentration ($mg\,kg^{-1}$) in Soil after 75 Days of Growth | | | Arsenic Content in Rice ($mg\,kg^{-1}$): Root | | | Stem | | | Leaf | | | Grain | | | Grain Yield (t/ha) | | |
|---|---|---|---|---|---|---|---|---|---|---|---|---|---|---|---|---|---|---|
| | $Zn_0$ | $Zn_1$ | Mean | $Zn_0$ | $Zn_1$ | Mean | $Zn_0$ | $Zn_1$ | Mean | $Zn_0$ | $Zn_1$ | Mean | $Zn_0$ | $Zn_1$ | Mean | $Zn_0$ | $Zn_1$ | Mean |
| $I_0$ | 4.48 | 2.81 | 3.64 | 8.93 | 8.87 | 8.90 | 6.97 | 6.87 | 6.92 | 6.98 | 6.86 | 6.92 | 1.03 | 0.94 | 0.98 | 4.01 | 4.84 | 4.42 |
| $I_1$ | 3.95 | 3.21 | 3.58 | 8.31 | 8.01 | 8.16 | 6.19 | 5.98 | 6.08 | 6.24 | 6.10 | 6.17 | 0.98 | 0.88 | 0.93 | 3.80 | 4.83 | 4.31 |
| $I_2$ | 3.53 | 1.98 | 2.75 | 7.71 | 7.15 | 7.43 | 6.35 | 5.14 | 5.24 | 5.78 | 5.49 | 5.63 | 0.96 | 0.81 | 0.88 | 3.65 | 4.19 | 3.92 |
| Mean | 3.98 | 2.66 | | 8.31 | 8.01 | | 6.17 | 5.99 | | 6.33 | 6.15 | | 0.99 | 0.87 | | | | |
| CD ($p = 0.05$) | | | | | | | | | | | | | | | | | | |
| I | | 0.66 | | | 0.29 | | | 0.37 | | | 0.35 | | | 0.29 | | | 0.29 | |
| Zn | | 0.82 | | | 0.19 | | | 0.28 | | | 0.31 | | | 0.23 | | | 0.42 | |
| I × Zn | | 1.42 | | | 0.37 | | | 0.48 | | | 0.53 | | | 0.39 | | | 0.73 | |

The highest accumulation of arsenic in roots as compared to stems, leaves, and grains might be due to its less mobility within the plant, which supports the results reported by Liu et al.[12 ] who showed the distribution of arsenic in plants was in the following order: Root > Stem > Leaf > Edible parts. Depression of rice growth seems to depend on the amount of arsenite in the soil and arsines damage the roots of rice, resulting inhibition of nutrient uptake[13]. Accumulation of arsenic in rice roots, stems, leaves, and grains was found to have decreased significantly with the application of either Zn or management of irrigation water or both; the lowest significantly in root (7.15 mg $kg^{-1}$), stem (5.14 mg $kg^{-1}$), leaf (5.49 mg $kg^{-1}$), and grain (0.81 mg $kg^{-1}$) with the application of Zn under intermittent ponding throughout the growth period of rice compared to other water management practices.

The highest amount of water was used in the treatment $I_0$ (160 cm $ha^{-1}$) followed by $I_1$ (124 cm $ha^{-1}$) and $I_2$ (102 cm $ha^{-1}$) for the growth of rice without affecting the yield of rice and without increasing the concentration of arsenic in both the soil and its uptake by rice plant. The grain yield of rice was recorded the highest in $Zn_1I_0$ treatment combination (4.98 t $ha^{-1}$), which was closely followed by 4.83 t $ha^{-1}$ in $Zn_1I_1$ treatment. Although, a slight decrease in the yield was recorded in $Zn_1I_2$ treatment (4.19 t $ha^{-1}$), but the concentration of arsenic in different parts of the rice plant and its buildup in the soil was significantly lower.

Considering the arsenic pollution problems, $Zn_1I_1$ treatment combination was superior in relation to marginal yield increase as well as reducing arsenic concentration in both soil and plants[14].

## BIOSKETCHES

**D.K. Das,** Professor in the Department of Agricultural Chemistry and Soil Science, has 25 years of experience in teaching on the undergraduate and postgraduate level, and research relating to soil chemistry, soil fertility, and plant nutrition. Prof. Das has published about 100 original research papers in national and international journals of repute and has also published six books from Kalyani Publishers, India. http://myprofile.cos.com/dkdas

**T.K. Garai** was a Senior Research Fellow of this project work funded by the Indian Council of Agricultural Research (ICAR), New Delhi, India

**S. Sarkar**, Professor in the Department of Agricultural Meteorology and Physics, has approximately 20 years of experience in teaching and research with particular reference to water management in agricultural production systems. Prof. Sarkar has published a good number of research papers in journals of national and international repute.

**Pintu Sur** is a Research Fellow in the Department of Agricultural Chemistry and Soil Science, Bidhan Chandra Krishi Viswavidyalaya, West Bengal, India. Sri Sur has published three research papers in journals of national and international repute.
